# Inflammatory Breast Diseases during Lactation: Health Effects on the Newborn—A Literature Review

**DOI:** 10.1155/2008/298760

**Published:** 2008-04-20

**Authors:** Achim Wöckel, Michael Abou-Dakn, Anna Beggel, Petra Arck

**Affiliations:** ^1^Department of Gynecology and Obstetrics, University Hospital Ulm, Prittwitzstraße 43, 89075 Ulm, Germany; ^2^Department of Gynecology and Obstetrics, St. Joseph Hospital, University Medicine Berlin - Charité, 12203 Berlin, Germany; ^3^Department of Psychoneuroimmunology, University Medicine Berlin - Charité, 13353 Berlin, Germany; ^4^Department of Medicine, Brain Body Institute, McMaster University Hamilton, ON, Canada L8S4L8

## Abstract

Breastfeeding-associated inflammatory breast diseases appear especially during the first twelve weeks postpartum and are the most common reason for early cessation of breastfeeding. It also becomes increasingly evident that these inflammatory mammary diseases are triggered or perpetuated in a large part by psychosocial stress. Immunological processes taking place during this cascade in the mammary gland and consequences for the breastfeed newborn are mostly yet unknown. This review summarizes insights from studies on modulation of cytokine levels in breast milk during inflammatory processes like milk stasis and mastitis systematically. It also gives an overview on possible pathological effects, which these cytokine changes in the breast milk might have on the newborn.

## 1. INTRODUCTION

### 1.1. Milk stasis and mastitis

The WHO suggests a six-month period of breastfeeding to all breastfeeding mothers [1]. Evidence-based advantages for mother and child result especially when children in this time are fed exclusively without any additional breast milk supplements [2]. Only 50% of women worldwide reach this goal [3]. The remaining mothers very often add supplements or cease breastfeeding completely because they suffer from inflammatory breast diseases like milk stasis or puerperal mastitis [4].

These breastfeeding-associated inflammatory breast diseases appear especially during the first twelve weeks postpartum and are the most common reason for an early cessation of breastfeeding [5]. Changes develop like a cascade [4–6]: first little erosions caused by the suckling of the infant lead to severe nipple and areolar pain. Pain anticipates an undisturbed breastfeeding relationship and leads to an insufficient emptying of the breast by the newborn. An insufficient emptying of the breast can subsequently lead to a stasis in the mammary alveoli. This milk stasis augments pain and opens intercellular junctions between the milk duct epithelial cells caused by a rise of intraductal pressure. Breast milk, then moving into the connective tissue, leads to a primary a sterile inflammation, generally followed by a secondary bacterial infection. In the worst cases, this results in a puerperal mammary abscess, which has to be surgically treated.

Immune mediators such as cell subsets or cytokines involved in this perpetuating inflammation in the mammary gland in humans are mostly unknown.

In veterinary medicine, a number of scientists work on the subject focussing on bovine mastitis in experimental studies, since bovine mastitis causes enormous economic damage in the dairy industry [7], and hence, the dairy industry has a vast interest in the development of analytical methods to identify animals at risk when symptoms are still inapparent. The goal of these research endeavors is to identify early risk markers, that is in milk or maternal serum. In this context, the somatic cell count (SCC/mL), bacterial count (colony-forming units [CFUs]/mL), ratio of milk phagocytes (mononuclear [Mphi] plus polymorphonuclear [PMN] cells) to lymphocytes (P/L index), and ratio of PMN to Mphi cells (PMN/Mphi index) and also the measurement of cytokines in milk could be used to identificate the inflammatory reactions in the mammary tissue [8]. Immunomodulating agents are also normally present in human milk in physiologically relevant quantities but there is a wide range of concentrations of different cytokines at each time during the first 12 weeks of lactation: IL-1: 15-400 pg/mL; IL-6: 15-1032 pg/mL; TNF-alpha: 15-2933 pg/mL; Prostaglandin E2: 10-9966 pg/mL; TGF-beta1: 43-7108 pg/mL; TGF-beta2: 208-57935 pg/mL [9].

### 1.2. Diseases of the breast during lactation and stress

It is generally believed that inflammatory breastfeeding-associated mammary diseases may be triggered or aggravated by psychosocial stress, as observed for example in veterinary medicine. Here, exposure to experimental stressors such as regrouping and relocation resulted into mastitis in animals, as described for lactating ewes [10]. In humans, clinical observations reveal similar correlations: mothers with breastfeeding-associated diseases (milk stasis and mastitis) report an increased stress perception, that is due to events in their social network in the weeks prior to the clinical symptoms [11]. Recent studies performed by the authors further support the alleged causality of stress perception and puerperal diseases in humans [12].

What are the possible pathophysiological mechanisms of stress-dependent breast diseases during lactation? The increased secretion of catecholamines in stressed mothers impairs the release and access of oxytocin to the mammary gland and the action of oxytocin on the secretory epithelium [13]. The release of oxytocin in response to stressful stimuli may reduce the availibility of this hormone at the suckling reflex. Stress also leads to higher levels of prolactine and thus to an increased synthesis of breast milk. The reduced release or impaired action of oxytocin and the coexistent higher proposal of breast milk cause an incomplete emptying of the alveoli and galactophorus ducts and lead to milk stasis.

Stressfull events may also cause immune supression in the mammary tissue. T-lymphocytes have regulatory functions or act directly on foreign antigens because of producing cytokines. T-lymphocytes are strongly involved in the defense against bacterial invasion during mastitis [14]. These cells are uniquely sensitive to soluble modulating factors, so it is likely that the neuroendocrine response in stress elaborates hormones and peptides that may have a major impact on cell mediated immunity [15]. The increase in maternal stress perception has recently been shown to cause a priming of the maternal immune system towards a proinflammatory, Th1-cytokine response (IL-1, IL-6, TNF-a, INF-y) instead of anti-inflammatory Th2-cytokines (IL-4, IL-5, IL-9 and IL-13) in the mammary tissue [12].

At birth the immune system of the neonate is primed towards a Th2 dominance. Within the first 2 years of life, the immune system is activated, probably via childhood infections, leading to a naturally occurring shift from Th2 to Th1 immunity. Intestinal mucosa is also premature in the first two years. Thus higher concentrations of proinflammatory Th-1-cytokines in the breast milk may lead to local and systemic immunological effects of the newborn [16].

### 1.3. Modified cytokine patterns of the breast milk and their effects on the child

Maternal stress perception is likely intimately linked to stress reactions of the newborn. Published data indicate that the offspring may develop somatic diseases [17], well described for atopic dermatitis, bronchitis, and allergic diseases in response to maternal stress perception [16, 18]. Most of these studies focus on imprinting such as maternal stress perception programming the child in utero, which is generally referred to as the fetal programming hypothesis. Surprisingly, to date, the period of lactation has received very little attention in this context. Here, it has been described that a long period of lactation minimizes the risk for infection of the offspring due to high levels of IgA in breast milk, which may protect the offspring from infectious diseases [19]. However, it still remains to be elucidated if and how maternal stress perception affects immune markers in the breast milk and whether such alterations may have consequences for the child's well being. Given the yet unexplained dramatic increase of chronic inflammatory diseases in children over the past 5 decades [20], the identification of a vicious cycle between stress perception, impaired breastfeeding/nutrition of the offspring and the onset of chronic diseases is urgently required and it is the aim of the present review to foster future research in this direction.

**Figure 1:**
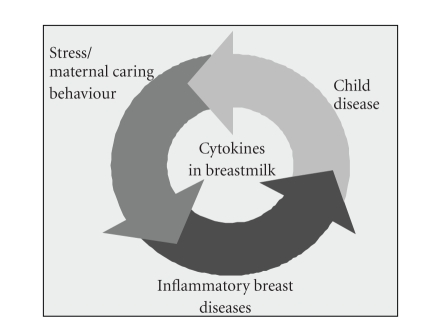
Vicious cycle of increased maternal stress perception, inflammatory breast diseases, and diseases of the newborn transmitted by changes in the cytokine pattern in breast milk.

If a major part of breast diseases, as proven in our own surveys, is caused by stress and at the same time the prevalence of these diseases is relatively high [12], a change in the constituents of breast milk would be imaginable. One possibility is a change in the cytokine profile in breast milk, which then might lead to diseases in the child (Figure 1). Further, recent studies predominately arising from rodents have elegantly shown that the epigenome of the developing fetus is sensitive to maternal nutrition, exposure to environmental toxins as well as to psychological stress [21]. It is postulated that exposure of the young pup to social behavior, such as maternal care, could affect the epigenome. Epigenetic alterations, which could have similar consequences as genetic polymorphisms, have been shown to arise from variations of maternal behavior and may account for differences in human behavior and possibly vulnerability to diseases later in life of the offspring. Hence, impaired breastfeeding could affect the growing offspring in a number of ways, that is via an altered immune cocktail in the breast milk. But also impaired maternal caring behavior due to discontinuation of breastfeeding may lead to effects on the psychological development and thus on the immune system of the newborn, because breastfeeding seems to be also very important for emotional bonding [22].

The aim of this systematic review was to show detectable changes of cytokines in breast milk during inflammatory processes of mammary tissue like mastitis and possible pathological effects of these mediators on the newborn caused by breastfeeding.

## 2. METHODOLOGY OF THE PRESENT REVIEW

**Figure 2:**
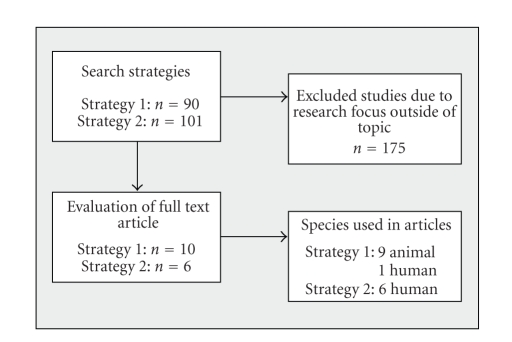
Flow-chart describing the process of selection and identification of literature.

In order to identify published evidence addressing the topics (1) cytokines detectable in inflammatory processes like a mastitis in breast milk and (2) pathological effects of cytokines in the breast milk on the newborn, literature databases (Medline, Embase) were searched. The following search strategies were developed to identify the publications most sensitively. *Search strategy 1:* (“mastitis” [MeSH Terms] OR mastitis [Text Word]) AND (“human milk” [Text Word] OR “milk, human” [MeSH Terms] OR “milk” [MeSH Terms] OR milk [Text Word]) AND (“cytokines” [MeSH Terms] OR cytokines [Text Word]); *search strategy 2:* (“cytokines” [MeSH Terms] OR cytokines [Text Word]) AND (“lactation” [MeSH Terms]) OR (“breast feeding” [TIAB] NOT Medline [SB]) OR “breast feeding” [MeSH Terms] OR LACTATION [Text Word]) AND (“newborn infant” [Text Word] OR “infant, newborn” [MeSH Terms] OR newborn [Text Word]). All articles published in German or English between 2002 until 2007 were included. This period was established to get the most current results of this field of research. Veterinary and human studies, experimental and clinical surveys were analysed. An overview on the process of research and selection is shown in Figure 2.

## 3. RESULTS

Searching according to the above-described key word strategy yielded a total of 191 publications (titles or abstracts): after selection of the literature, 16 publications remained (see Figure 2), 10 of which referred to the first topic, 6 focussing on the second topic. 175 of the publications addressed none of the two topics of interest and were excluded: most of these experimental or observation-studies described changes of cytokines in blood (and not in breast milk) during mastitis, which was not the focus of this review.

Results from the first search strategy are presented in Table 1. All identified studies [23–32] describe an increase of predominately proinflammatory cytokines in milk or peripheral leukocytes in response to experimental challenge set by artificial infection with different germs. Stress perception has not been included in the design of these studies.

Results from the second search strategy are presented in Table 2. Here, we identified 5 cohort studies [33–37] where cytokine levels in breast milk were linked to different alterations or diseases in the offspring. During revision and selection of the literature, we also identified one further publication [38], which especially examined the influence of cytokine-patterns on the incidence of allergies in the child. In this publication, a correlation between these variables could not be established. Thus, a change of cytokines in breast milk may not have an influence on the incidence or progression of allergies.

## 4. CONCLUDING REMARKS

An increase of cytokines in breast milk has been reported from different stages of maternal lactation: on the one hand, maternal diseases during pregnancy—like pre-eclampsia or allergies—can lead to a rise in cytokines of breast milk [39]. In addition, the systematic review of the literature performed here showed a modulation of cytokine levels in breast milk during inflammatory chest diseases during lactation. As there are mostly only animal studies available, it has to be investigated in humans if and to which extent levels of cytokines are modulated in breast milk. In addition, this review revealed that an imbalance of cytokines in breast milk may have severe consequences for the child, which in turn affects the child's development. However, the studies summarized here with regard to the two topics focussed on different cytokines and in different species. Future work is needed to clear if and how these observations can be translated into clinical significance. Further, none of these studies included stress perception as a possible trigger for cytokine imbalances in the breast milk. Nonetheless, it still remains to be elucidated how stress perception may trigger inflammatory events of the breast. Further, one may easily envision that mastitis and the related impaired breastfeeding ability itself are potent stressors, which may additionally aggravate the clinical symptoms. Thus, research endeavours should focus on the identification of markers, preferably immune markers, prior to the onset of clinical symptoms. To date, the relationship between stress and mastitis is supported by own observations [12] and will have to be confirmed in larger collectives. However, it is nowadays well accepted that high stress perception can alter immune hemostasis and render the individual, both adults and newborn, less resistant to infectious diseases. It has been suggested that this is due to increased levels of corticosteroids which can effect the functions, pattern, and numbers of leucocytes and thus increase the hostçs susceptibility to infections [40].

What are possible pathophysiological mechanisms for the shown increase of interleukines in breast milk during inflammatory processes? The proportion of T cells in mammary tissue normally declines during lactation and the number of T cell subsets (CD4, CD8, and WC1-T cells in ruminants) varies significantly during this period. The proportions of several cell populations (CD2, CD4, CD8, MCHC II) are lower in milk than in blood following parturition, while the proportion of WC1+ cells is higher in milk [40]. But an increase of plasma cortisol (i.e., caused by stress) has been shown to decrease the number of circulating lymphocytes. The number of T cells in blood declines after the rise of serumcortisol, but expression of adhesion molecules on these cells is not affected. This suggests that glucocorticoids can enhance migration of T cells from blood into mammary tissues [41]. This might be important to avert inflammatory reactions and to perpetuate the secretion of cytokines in the gland and the breast milk.

What kind of biological mechanism could be made responsible for that? So on the one hand, a rise in cytokines of breast milk is useful to activate a mechanism of maternal self-defence against infectious processes in the glandular tissue [42]. On the other hand, a rise in cytokines in breast milk could be useful in breastfed infants in order to activate or stimulate their immunity [43]. It is possible though that a permanent oversupply of cytokines (i.e., triggered by high maternal stress perception) leads to an excessive stimulation/threat of the child immune system and subsequently to the onset of diseases. This hypothesis is supported by results by Elmlinger et al. [44] and Ustundag et al. [45] who showed that breast milk from mothers who delivered preterm children contained lower levels of cytokines compared to breast milk from mothers of term children. The proposal of cytokines in maternal breast milk seems to adapt to the requirement of the newborn and acts in accordance with development status of the immune system. Probably the immune system of the newborn is fragile for disruptive factors also like changes of cytokine-concentrations mediated my breastfeeding.

The review shows evidence of increased cytokines in breast milk during inflammatory processes like mastitis and possible pathological effects of these higher cytokine-concentrations on the newborn. A correlation between these consequences on state of health and special interleukins in breast milk could not be detected in the current literature and should be investigated in further studies.

## Figures and Tables

**Table 1:** Key findings on immune alteration in breast milk, identified upon search for the topic “cytokines detectable in inflammatory processes like a mastitis in breast milk.”

Publication	Animal study	Human study	Key finding
Dernfalk et al. [23]	+		The quantification of enhanced proinflammatory cytokines IL-1beta, IL-6, and TNF-*α* in bovine whey or milk samples is indicative for an acute inflammatory response of mammary tissue.
Bannerman et al. [24]	+		Persistingly increased levels of TGF-*α*, -ß1, and -ß2 in milk were evident upon infection with *S. aureus*.
Lee et al. [25]	+		Inflammatory cytokines (interleukin (IL)-6, IL-8, IL-12, granulocyte macrophage-colony stimulating factor (GM-CSF), tumor necrosis factor TNF-*α*, and interferon (IFN)-*γ*, secreted by somatic cells present in the breast milk) were characterized by real-time polymerase chain reaction (PCR) in dairy cows upon experimental challenged with either *E. coli* or *S. aureus.*
Bannerman et al. [26]	+		Systemic and localized bovine innate immune responses to intramammary infection with *P. aeruginosa* were characterized and increased levels of IL-8, TNF-*α*, IL-10, and IL-12 were detected. Elevation of these cytokines was not sustained for longer than a 24-hour period.
Chockalingam et al. [27]	+		Analysis of whey samples derived from *E. coli*-infected quarters revealed an increase of TGF-*α*, -ß1, and -ß2.
Rambeaud et al. [28]	+		*S. uberis* challenge induced local production of TNF-*α*, IL-1ß, and IL-8 in mammary tissue.
Alluwaimi et al. [29]	+		Transcriptional activity of bovine cytokines IL-12 and TNF-*α* levels was significantly elevated upon experimental *S. aureus* infection. Levels of IL-2 were decreased. IL-12 and TNF-*α* levels were significantly elevated at 24 hours post-infectionem (pi) followed by sharp decrease at 32 hours pi.
Persson Waller et al. [30]	+		Intramammary infusion of endotoxin from *E. coli* in cows resulted in neutrophil increase in afferent and efferent supramammary lymph nodes. Concentrations of IL-8 increased in lymph nodes. TNF-*α* levels increase in lymph nodes and milk. The levels of IL-1ß increased in milk, but were not detected in lymph nodes. Interferon-*γ* was undetectable.
Riollet et al. [31]	+		IL-1*α*, IL-1ß, IL-6 and TNF-*α*, IL-10, and IL-12 mRNA were synthesized in cells derived from infected mammary glands, whereas neither IL-2 nor IL-4 mRNA could be detected.
Prgomet et al. [32]		+	Cell culture models were established, where milk somatic cells and peripheral leukocytes were cultured and activated with lipopolysaccharide (LPS). Via real time RT-PCR, increased cytokine mRNA levels could be detected for TNF-*α*, IL-6, and IL-1ß, which persisted longer in peripheral leukocytes compared to milk somatic cells.

**Table 2:** Key findings on immune alteration in breast milk, identified upon search for the topic “pathological effects of cytokines in the breast milk on the newborn.”

Publication	Animal study	Human study	Key finding
Zanardo et al. [33]		+	Levels of IL-1ß are significantly increased in colostrum from breast-feeding mothers whose infants have hyperbilirubineamia.
Moore et al. [34]		+	Levels of IL-7 in breast-milk, sensitive to seasonal influences, may mediate thymus function of the newborn.
Prokešová et al. [35]		+	Allergic mothers exhibit markedly higher IL-10 levels in breast milk compared to healthy mothers.
Rigotti et al. [36]		+	Lower levels of TGF-ß1 are present in mature milk of allergic mothers.
Bryan et al. [37]		+	Breast milk from mothers of infants hospitalized with bronchiolitis had significantly higher levels of IL-2 and IL-10 compared with milk from mothers of postpartum age-matched healthy controls.
Böttcher et al. [38]		+	There was no association between levels of IL-4, -5, -6, -8, -10, -13, -16, IFN-*γ*, TGF-ß1, -ß2, in the breast milk of mothers whose infants developed allergic symptoms or salivary IgA levels during the first 2 years of life. Thus, differences in the composition of cytokines and chemokines in breast milk did not, to any major degree, affect the development of atopic symptoms nor salivary IgA antibody production during the first 2 years of life.
